# Treatment of toxic epidermal necrosis lesions with ovine forestomach matrix

**DOI:** 10.1093/jscr/rjaf742

**Published:** 2026-02-06

**Authors:** Rodrigo Dornelles, Jessica Simon, Sabraj O C Dutra

**Affiliations:** Plastic Surgery, Hospital Beneficência Portuguesa de São Paulo, Rua Maestro Cardim 69, São Paulo 01323-000, Brazil; Aroa Biosurgery Limited, 64 Richard Pearse Drive Auckland, 2022, New Zealand; Plastic Surgery, Hospital Beneficência Portuguesa de São Paulo, Rua Maestro Cardim 69, São Paulo 01323-000, Brazil

**Keywords:** Stevens-Johnson syndrome, toxic epidermal necrolysis, extracellular matrix, ovine forestomach matrix, re-epithelialization

## Abstract

Stevens–Johnson syndrome and toxic epidermal necrosis (TEN) are used to describe a spectrum of acute mucocutaneous reactions primarily affecting the skin and mucous membranes, resulting in painful blisters and epidermal detachment. Swift diagnosis followed by management of the damaged epidermis is vital to prevent further infection, which if left untreated can become life-threatening. We report a case of TEN in a 42-year-old female who presented with 55% epidermal loss. Over the course of 41 days, the affected regions were treated with ovine forestomach matrix (OFM) grafts, which led to epithelial restoration of the lesions in 1–2 weeks with good functional and cosmetic outcomes. This case is the first documented use of OFM in the treatment of TEN lesions.

## Introduction

Stevens–Johnson syndrome (SJS) and toxic epidermal necrosis (TEN) describe a spectrum of acute, adverse reactions that affect the epidermis and mucous membranes. SJS is defined as <10% body surface area (BSA) detachment, while TEN is >30% BSA detachment, with an overlap of SJS/TEN between 10% and 30% [1–3]. Initial presentation of SJS/TEN may include fever, headaches, coughing, and malaise [4], followed by rapid deterioration and characteristic mucocutaneous lesions and large areas of epidermal detachment [2]. It is believed that the adverse reactions are drug-induced, and underlying infection may play a role.

If not quickly resolved, SJS/TEN can lead to a medical emergency, with potentially severe morbidity and mortality. Once the diagnosis has been confirmed, and any offending drugs are halted, treatment may focus on addressing the epidermal lesions [4, 5]. Standard wound care may be augmented with advanced biologic dressings to promote rapid restoration of epidermal coverage in SJS/TEN and other related bullous diseases (e.g. pemphigus vulgaris, PV). Here, we describe a case of Lamotrigine-induced TEN in a patient who presented with extensive epidermal loss. The patient was successfully treated with the biologic dressing, ovine forestomach matrix (OFM).

## Case report

**Figure 1:**
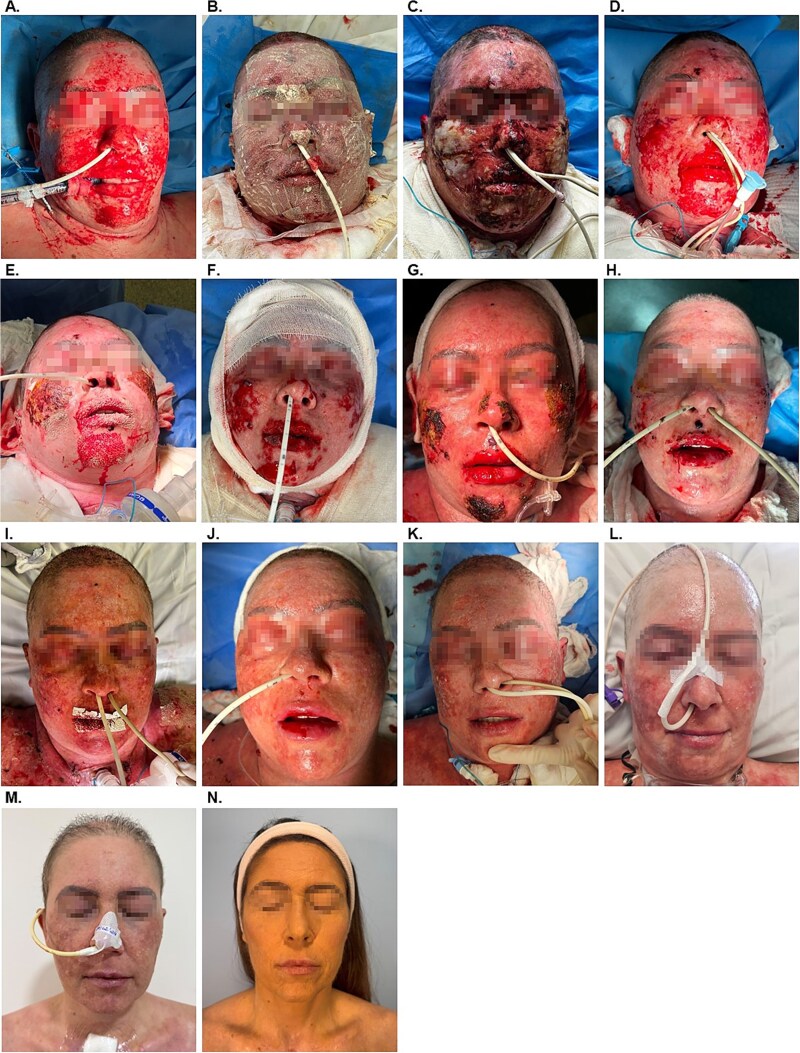
Representative images of the treatment progression. (A) Day 1 on admission to the ICU. (B) Day 2, post application of antimicrobial OFM. (C) Day 4, post dressing removal. (D) Day 8 on removal of the dressing and post light debridement. (E) Day 10. (F) Day 13, post light debridement. (G) Day 17. (H) Day 19. (I) Day 22. (J) Day 25. (K) Day 31. (L) Day 38. (M) Day 66. (N) Six month follow-up.

A 42-year-old woman with a history of depression presented to the ICU at Hospital Beneficência Portuguesa de São Paulo with lesions and epidermal loss to the face, head, neck, torso, pubis and parts of the upper extremities. The patient had been prescribed the anticonvulsant Lamotrigine for the management of depression, which likely triggered the TEN. Though rare, this reaction to Lamotrigine is well documented [6, 7]. The patient had been symptomatic for 2–3 weeks, with lesions developing and worsening. The face was the most affected region (Fig. 1A). Upon admission, no prior interventions had been undertaken.

On admission, acute infection was suspected, and the patient was started on systemic antibiotic therapy, including: piperazine (4 g oral daily for 2 days), teicoplanin (400 mg intravenous every 12 hours for three doses then 6 mg/kg intravenous daily), and Tazocin® (4.5 g intravenous every 6–8 hours), in addition to human immunoglobulin (0.4 g/kg continuous intravenous for 5 days) and anticoagulation protocol with Clexane® (40 mg subcutaneous once daily). Nutritional support was initiated, with an emphasis on protein balance and vitamin supplementation.

Given the suspected acute infection, a treatment plan was developed using antimicrobial OFM (Endoform™ Antimicrobial, Aroa Biosurgery Limited, New Zealand). The worst affected areas of the face were to be addressed first, staging treatment of the mild lesions in other parts of the body. The patient underwent the first application of antimicrobial OFM to the facial region on Day 2 (Fig. 1B), with evaluation scheduled for every 2 days. On Day 4 (Fig. 1C), dressings were removed and the patient showed signs of improvement and partial healing, with a reduction in inflammation and early signs of healing. A second application of antimicrobial OFM was performed on Day 4. On post-operative Day 8, dressings were removed and a gentle debridement performed (Fig. 1D). As the prior culture swab had returned positive for *P. aeruginosa,* a third application of antimicrobial OFM was undertaken. The patient received a fourth and fifth application at Days 10 (Fig. 1E) and 13 (Fig. 1F). On Day 17 (Fig. 1G) inflammation was markedly reduced. Targeted treatment with OFM was made to areas of the face including the lips, chin, and cheeks on Days 17 (Fig. 1G), 19 (Fig. 1H), and 22 (Fig. 1I). Given the positive response, OFM treatment was expanded to include affected regions of the torso and pubis beginning on Day 17. The patient was evaluated again on Days 25 (Fig. 1J) and 31 (Fig. 1K). The final inpatient evaluation was on Day 38 (Fig. 1L), where the patient was awake and in isolation. After removal of dressings, the patient showed near complete re-epithelialization of the facial wounds. Similarly, lesions to the upper extremities, torso and pubis (not shown) had progressed to near complete epithelialization, and minor areas were allowed to heal through secondary intention. The patient was discharged from the ICU by the plastic surgery team by Day 66 (Fig. 1M). Long-term follow-up at 6 months (Fig. 1N) demonstrated excellent cosmesis of the affected areas.

## Discussion

SJS/TEN are relatively rare conditions affecting an estimated 3–4 per million worldwide [8], however, there is no standardized method to treat the life-threatening lesions. Various wound care interventions, including biologic xenografts and biosynthetic skin substitutes have been described [9], with a primary goal of restoring epidermal coverage to prevent infection.

While OFM-based devices have been widely used in the management of acute and chronic wounds [10, 11], the current case study describes the first reported use of OFM to treat SJS/TEN lesions. Research has described the successful use of OFM to treat extensive epidermal loss resulting from the related disease, PV [12]. In this case report, the authors describe complete re-epithelialization of the lesions within one week and a dramatic resolution of patient pain [12]. While the mechanism of OFM-induced healing seen in both PV and SJS/TEN is unknown, it may be linked to anti-inflammatory proteins, including tissue inhibitors of matrix metalloproteinases (TIMPs) present in OFM [13]. This is particularly relevant as both conditions involve inflammation of the affected tissues. For example, Arafat et al. [14] showed significantly increased concentrations of matrix metalloproteinases, MMP8 and MMP9, and a decreased concentration of TIMP-1 in SJS lesions. The elevated MMP-to-TIMP ratios and high MMP activity suggest an imbalance in MMP regulation, which would account for the inflammation and slow rate of epithelialization of SJS/TEN lesions [14]. Interestingly, in addition to the known anti-inflammatory components present in OFM, studies have shown that OFM is a potent broad-spectrum inhibitor of tissue proteases, including MMPs [15]. The success of treating SJS/TEN and PV with OFM may be linked to this functionality.

Treating epidermal lesions is critical to reduce the risk of infection in SJS/TEN patients. The current case describes the successful use of OFM to treat epidermal detachment in SJS/TEN. We recommend that further studies investigate the use of OFM for management of SJS/TEN and related bullous diseases.
